# C-reactive protein-complement factor H axis as a biomarker of activity in early and intermediate age-related macular degeneration

**DOI:** 10.3389/fimmu.2024.1330913

**Published:** 2024-04-03

**Authors:** Lena Giralt, Marc Figueras-Roca, Beatriz De Luis Eguileor, Barbara Romero, Javier Zarranz-Ventura, Socorro Alforja, Francisca Santiago, Jennifer Bolaños, Francisco Lozano, Marina Dotti-Boada, Anna Sala-Puigdollers, Paula Dura, Jordi Izquierdo-Serra, Oliver Valero, Alfredo Adan, Alex Fonollosa, Blanca Molins

**Affiliations:** ^1^ Institut Clínic d’Oftalmologia (ICOF), Hospital Clínic, Barcelona, Spain; ^2^ Department of Ophthalmology, Biocruces Bizkaia Health Research Institute, Cruces University Hospital, University of the Basque Country, Barakaldo, Spain; ^3^ Group of Ocular Inflammation: Clinical and Experimental Studies, Institut d’Investigacions Biomèdiques Agustí Pi I Sunyer (IDIBAPS), Barcelona, Spain; ^4^ Servei d’Immunologia, Centre de Diagnostic Biomèdic, Hospital Clínic Barcelona, Barcelona, Spain; ^5^ Group of Immunoreceptors of the Innate and Adaptive Systems, Institut d’Investigacions Biomèdiques Agustí Pi I Sunyer (IDIBAPS), Barcelona, Spain; ^6^ Departament de Biomedicina, Facultat de Medicina, Universitat de Barcelona, Barcelona, Spain; ^7^ Servei d’Estadística, Universitat Autònoma de Barcelona, Barcelona, Spain; ^8^ Department of Retina, Instituto Oftalmológico Bilbao, Bilbao, Spain

**Keywords:** age-related macular degeneration, inflammation, complement factor H, C-reactive protein, retina, biomarker

## Abstract

**Purpose:**

To determine and compare the serum levels of complement Factor H (FH), monomeric C-Reactive Protein (mCRP) and pentameric C-Reactive protein (pCRP) in patients with age-related macular degeneration (AMD) and to correlate them with clinical, structural and functional parameters.

**Methods:**

Cross-sectional observational study. One hundred thirty-nine individuals (88 patients and 51 healthy controls) from two referral centers were included and classified into three groups: early or intermediate AMD (n=33), advanced AMD (n=55), and age and sex matched healthy controls (n=51). Serum levels of FH, mCRP, and pCRP were determined and correlated with clinical and imaging parameters.

**Results:**

Patients with intermediate AMD presented FH levels significantly lower than controls [186.5 (72.1-931.8) µg/mL vs 415.2 (106.1-1962.2) µg/mL; p=0.039] and FH levels <200 µg/mL were associated with the presence of drusen and pigmentary changes in the fundoscopy (p=0.002). While no differences were observed in pCRP and mCRP levels, and mCRP was only detected in less than 15% of the included participants, women had a significantly higher detection rate of mCRP than men (21.0% vs. 3.8%, p=0.045). In addition, the ratio mCRP/FH (log) was significantly lower in the control group compared to intermediate AMD (p=0.031). Visual acuity (p<0.001), macular volume (p<0.001), and foveal thickness (p=0.034) were significantly lower in the advanced AMD group, and choroidal thickness was significantly lower in advanced AMD compared to early/intermediate AMD (p=0.023).

**Conclusion:**

Intermediate AMD was associated in our cohort with decreased serum FH levels together with increased serum mCRP/FH ratio. All these objective serum biomarkers may suggest an underlying systemic inflammatory process in early/intermediate AMD patients.

## 1 Introduction

Age-related macular degeneration (AMD) is the leading cause of irreversible vision loss among the elderly in developed countries and carries a significant socioeconomic burden for health systems and family members due to its chronicity and tendency to progression. It is a chronic degenerative disease that can be divided into early, intermediate and advanced stages (1). Early AMD manifests as retinal pigment irregularities and drusen; intermediate AMD has confluent groupings of drusen of medium and large size, and a higher risk of progressing to advanced AMD. Advanced AMD is characterized by geographic atrophy or choroidal neovascularization which can lead to blindness.

AMD is a multifactorial disease, where both genetic and environmental factors converge. Local inflammatory and immune-mediated mechanisms are pivotal in the pathophysiology of AMD (2, 3), and inflammatory proteins and components of the complement system are present in ocular drusen (2, 3). A genetic influence on the AMD risk has also been demonstrated for complement-related genes (4). There is a strong genetic susceptibility associated with the complement factor H (*CFH*) locus (5). In particular, a common polymorphism in the *CFH* gene (rs1061170; c.1277T>C, p.Tyr402His) accounts for the strongest genetic risk factor for AMD (6–8). This polymorphism results in a decreased binding affinity to numerous retinal components, leading to a decreased inhibitory effect of factor H (2).

C-reactive protein (CRP), an acute phase-reactant and active regulator of the innate system, is considered a biomarker of inflammation. Similar to cardiovascular disease, small increases in CRP are associated with AMD progression (9). In plasma, CRP exists as a 115 kDa cyclic pentamer (pCRP). Nevertheless, this pentameric circulating form can dissociate into its monomeric subunits of 23 kDa when bound to damaged or apoptotic cell membranes (10). This monomeric form (mCRP) has different antigenicity and biological properties representing the tissue-associated proinflammatory form. mCRP recruits C1q to the surface of damaged cells and thereby initiates complement activation resulting in the formation of C3 convertases and C3b surface deposition and inflammation (11). mCRP also recruits complement inhibitors, such as C4b-binding protein (C4BP) and FH, which both block complement progression at the level of C3 and inhibit inflammation. Both C1q, C4BP, and FH compete for mCRP binding, and this competition adjusts the local balance of activation and inhibition (12, 13). In the context of AMD, mCRP induces a proinflammatory phenotype in the retinal pigment epithelium (RPE) and contributes to the disruption of the outer retinal-blood barrier (oBRB) *in vitro* (13, 14). Both CRP forms have been detected in ocular drusen, but it is unclear the relative contribution of local and systemic CRP to the ocular CRP pool. The pCRP from choroidal circulation may reach the subRPE and locally dissociate into the proinflammatory mCRP form, activating the downstream complement system pathway on-site (15). Alternatively, mCRP may be more likely generated in the surface of activated choroidal endothelial cells and trespasses the oBRB, inducing a chronic inflammatory state in the RPE-Bruch’s membrane complex. Further, FH binds mCRP to dampen its proinflammatory activity. Notably, FH from AMD patients carrying the “risk” *CFH* polymorphism (rs1061170; p.Tyr402His), displays impaired binding to mCRP, and therefore proinflammatory effects of mCRP remain unrestrained (13). Factor H-like protein 1 (FHL-1), a truncated form of FH that contains the rs1061170 polymorphism, also plays a significant regulatory role in complement homeostasis in the outer retina and interacts with mCRP (16).

Despite the compelling evidence that local mCRP and FH are involved in the progression of AMD, it is unknown whether systemic mCRP is altered in AMD patients and whether the systemic FH-mCRP axis could be a biomarker of AMD activity. This work aims to investigate further the role of systemic FH-mCRP axis as a potential biomarker of disease severity in AMD patients.

## 2 Methods

### 2.1 Study design, patient population, and data collection

This was a cross-sectional, multicenter observational study of patients with AMD in two Spanish tertiary referral hospitals (Hospital Clinic of Barcelona and Hospital Universitario Cruces) between January 2020 and February 2024.

Inclusion criteria were age >55 years-old and AMD diagnosis according to eye fundus examination. Exclusion criteria included ocular media opacity, any history of cancer in the past 10 years, cardiovascular disease with organic complication during the last year, diabetes mellitus with poor glycemic control (glycated hemoglobin >7.5%) or end-organ damage, systemic or ocular autoimmune disease, concomitant retinopathy other than AMD, myopia >6 diopters or axial length >26 mm, and current treatment with anti-inflammatory drugs (corticosteroids, NSAIDs, immunosuppressors, biologic drugs).

Patients were classified into early (drusen >63 microns with no pigmentary changes) or intermediate AMD (drusen>125 microns and/or pigmentary macular changes) and advanced AMD (geographic atrophy, choroidal macular neovascularization, subretinal fibrosis). For statistical analysis, early and intermediate AMD were merged into a unique group. A control group was recruited (healthy subjects aged >55 without exclusion criteria) and matched by age and sex.

Patient information was anonymized at the time of inclusion. All participants were of Caucasian origin and provided written informed consent. The research followed the tenets of the Declaration of Helsinki and the Hospital Clínic of Barcelona Institutional Review Board (IRB) approved this study according to local and national IRB guidelines (HCB/2017/0558).

All patients underwent detailed ophthalmological exploration including visual acuity assessment, intraocular pressure measurement, anterior segment biomicroscopy, fundus examination, color and autofluorescence Optos Ultra Wide-Field retinography (Optomap, Dunfirmline, UK), and macular Spectral-domain optical coherence tomography (SD-OCT : Cirrus HD-OCT 5000, Carl Zeiss Meditec, Dublin, CA, US).

### 2.2 Genotyping

Genomic DNA was purified from ethylenediaminetetraacetic acid (EDTA)-treated peripheral blood from 112 individuals using the QIAamp DNA Blood Mini Kit (Qiagen, Venio, The Netherlands). For genotyping of the rs1061170 CFH gene polymorphism (Tyr402His), DNA samples were subjected to real-time (RT)-PCR in a LightCycler^®^ 480 Multiwell plate 96 instrument (Roche, Life Sciences) using TaqMan SNP Genotyping assay number C_8355565_10 (ThermoFisher Scientific), following manufacturer’s instructions.

### 2.3 Determination of systemic levels of FH, pCRP and mCRP

pCRP and FH levels were quantified from peripheral serum samples using commercial ELISA kits specific for human CRP (high-sensitivity CRP [hsCRP], IBL International GmbH) and FH (AssayPro human FH ELISA kit), respectively following the manufacturer’s instructions.

Serum mCRP was detected by a customized ELISA assay following the protocol described by Zhang et al. with some modifications (17). In brief, mouse anti-human CRP mAb CRP-8 (Sigma-Aldrich, C1688) was immobilized as capture antibody at 1:1.000 in coating buffer (10 mM sodium carbonate/bicarbonate, pH 9.6) overnight at 4°C. This commercially available monoclonal antibody has been reported to specifically recognize mCRP, but not pCRP (18). After washing three times for 2 minutes with TBS, non-specific binding sites were blocked with filtered 1% BSA-TBS for 1 hour at 37°C. Samples diluted 1:100 in blocking buffer were then added for 1 hour at 37°C. Then, samples were washed again and incubated with sheep anti-human CRP polyclonal antibody (MBS223280, MyBioSource) at 1:5000 in blocking buffer for 1h at room temperature (RT), prior incubation with a HRP-labeled donkey anti-sheep IgG (Abcam) at 1:10000 in blocking buffer for 30 min at RT. Signaling was detected with a VersaMax Microplate Reader and The OD value of each sample was calculated as OD450–OD570 nm. A standard curve was prepared by serial dilutions of mCRP (0-100 ng/mL) obtained by urea-chelation of pCRP (Calbiochem) in blocking buffer (1% BSA-TBS) in the presence of reference diluted sera (1:100).

### 2.4 Statistical analysis

Bivariate tests were performed among groups and the biomarkers with the rest of variables as follows: for qualitative variables the appropriate test for homogeneity of discrete distributions (Chi-square test or likelihood ratio test) was used depending on compliance with the application criteria; for quantitative variables, the application conditions of the different tests were analyzed (Shapiro-Wilk normality tests and Levene variance homogeneity tests). The appropriate model based on compliance with the application criteria (ANOVA or Kruskal-Wallis test) was used. The mCRP/FH and pCRP/FH ratios were log-transformed prior to statistical testing to accommodate its deviation from normality. P-values for pairwise comparisons were adjusted for multiplicity using Bonferroni’s correction. The analysis was performed using SAS 9.4 (SAS Institute Inc., Cary, NC, USA). The significance level was set to 0.05.

## 3 Results

A total of 139 individuals were recruited: 55 with advanced AMD, 33 with early/intermediate AMD, and 51 control healthy subjects. **Table 1** shows the descriptive analysis for each group. The mean ± SD age was 72.47 ± 7.37 years old in the control group, 75.21 ± 7.77 in the early/intermediate AMD and 79.40 ± 7.34 in the advanced AMD group (p<0.001). Ninety-three females (66.9%) and 46 males (33.1%) were included, but no differences between groups were observed. Mean follow-up was 35.37 ± 28.1 months in the early/intermediate AMD group and 49.84 ± 35.55 months in the advanced AMD group. Patients with advanced AMD presented higher prevalence of cardiovascular risk factors (i.e. hypertension, hyperglycemia, metabolic syndrome, and/or diabetes mellitus), although the difference was not statistically significant (p=0.192). Genotyping of the rs1061170 SNP in *CFH* gene revealed that the “risk” allele (homozygosity) was present in 13.5% of control subjects, 15% of intermediate AMD group, and 19% of advanced AMD patients.

**Table 1:** Descriptive analysis of control, early/intermediate and advanced AMD patients.

	Control	Early/Interm. AMD	Advanced AMD	p-value
**Age (years)** *Mean ± SD*	72.47 ± 7.37^a^	75.21 ± 7.77^b^	79.40 ± 7.34^b^	<0.001**
Sex (N, %)				
Female Male	31 (33.3)	26 (28)	36 (38.7)	0.221
	20 (43.5)	7 (15.2)	19 (41.3)	
**CRF (N, %)**	17 (24.3)	16 (22.9)	37 (52.9)	0.192
**Evol. time (months)** *Mean ± SD*		35.37 ± 28.14	49.84 ± 35.55	0.101
**BCVA (logMAR)** *Median (min.- max.)*	0.05 (0.00-0.30)^a^	0.15 (0,00-1.00)^b^	0.40 (0.0-3.00)^c^	<0.001**
**IOP (mmHg)** *Mean ± SD*	16.93 ± 3.02	16.42 ± 2.17	15.98 ± 3.34	0.311
Fundus exam (N,%)				
Drusen and/or Pigmentary changes Inactive NVM with drusen Other (Atrophy, Fibrosis, Active NVM, PED)		33 (100)^a^		<0.001**
			12 (21.8)^b^	
			43 (78.2)^b^	
Macular Cube				
Foveal thickness (µm) *Median (min-max.)* Macular volume (mm^3^) *Mean ± SD* Choroidal thickness (µm) *Median (min-max.)*	265 (226-310)	256 (201-306)	240 (93-479)	0.034*
	10.08 ± 0.46^a^	10.22 ± 0.53^b^	9.55 ± 0.95^b^	<0.001*
	190 (100 – 349)^ab^	226 (127 - 330)^a^	170 (60 - 354)^b^	0.007*
Biomarkers				
mCRP (ng/mL) *Median (min-max)*	1.00 (1.00-3007)	1.00 (1.00–1305)	1.00 (1.00–5852)	0.552
pCRP (µg/mL) *Median (min- max)*	1.68 (0.05-18.13)	1.39 (0.10-15.68)	1.49 (0.06-15.57)	0.958
Ratio mCRP/pCRP (log) *Median (min- max)*	-0.52 (-2.52-7.12)	-0.23 (-2.75-6.70)	-0.07 (-2.75-8.83)	0.453
FH (ng/mL) *Median (min- max)*	415.23^a^ (106.13-1962.2)	186.47^b^ (72.11-931.78)	380.27^a,b^ (5.39- 2680)	0.039*
Ratio mCRP/FH (log) *Median (min- max)*	-5.95 (-7.58-3.22)	-5.15 (-6.84-2.35)	-5.72 (-7.89-3.79)	0.031*
SNP Y402H (%)				
*TT* *CT* *CC*	34.1	29.3	36.6	0.975
	34.0	30.2	35.8	
	27.8	27.8	44.4	

SD, Standard deviation; CRF, cardiovascular risk factors (high blood pressure, cholesterol, diabetes, obesity); Evol., evolution; BCVA, Best Corrected Visual Acuity; IQR, Interquartile range; Max., maximum; Min., minimum; IOP, intraocular pressure; NVM, neovascular membrane; PED, pigment epithelium detachment; mCRP, monomeric C-reactive Protein; pCRP, pentameric C-reactive Protein; FH, complement Factor H; SNP, single nucleotide polymorphism. Pairwise comparisons: values not sharing any letter are significantly different at the 5% level of significance. Bold values are statistically significant values.

BCVA (logMAR) was significantly worse in both AMD groups: 0.05 (0.00 – 0.30) in the control group, 0.15 (0.00 – 1.00) in the early/intermediate and 0.40 (0.00 – 3.00) in the advanced group (p<0.001). Likewise, most advanced stages presented more fundus alterations (p<0.001), as shown in **Table 1**. The early/intermediate AMD group presented drusen (n=26), pigmentary changes (n=2) or both (n=5). The advanced AMD group presented drusen (n=5), pigmentary changes (n=5), both (n=2), atrophy (n=12), fibrosis (n=9), active neovascular membrane (n=14) or pigmentary epithelium detachment (n=8).

Macular volume was significantly higher in early/intermediate AMD (10.22 ± 0.53 mm3) compared to control (10.08 ± 0.46 mm3) and advanced AMD groups (9.55 ± 0.95 mm3) (p<0.001). Similarly, foveal thickness was thinner in AMD groups: early/intermediate AMD 256 (201-306) µm, advanced AMD 240 (93-479) µm, control 265 (226-310) µm, (p=0.034). Mean choroidal thickness was significantly higher in early/intermediate AMD 226 (127-330) µm compared to advanced AMD 170 (60-354) µm (p=0.023).

### 3.1 Systemic levels of CRP conformations in AMD

Serum levels of pCRP, measured by hsCRP ELISA did not differ among groups: 1.39 (0.10–15.68) µg/mL in the early/intermediate AMD group, 1.49 (0.06–15.57) µg/mL in the advanced AMD group and 1.68 (0.05 – 18.13) µg/mL in the control group (p=0.958). Likewise, high levels of pCRP (defined as pCRP>3 µg/mL) were not associated with any demographical, ophthalmological, and structural OCT parameter. Only, in early/intermediate AMD, high levels of pCRP were associated with thinner choroid (p=0.024). The mCRP levels were below the detection limit in 85% of subjects. Indeed, only 9.8% of control subjects had detectable mCRP levels, while this percentage was higher in intermediate and advanced AMD patients (15.2% and 18.2%, respectively). Bivariate analysis showed an association of mCRP with pCRP, and high mCRP levels (>1 ng/ml) were associated with high pCRP levels (>3 µg/mL) (p=0.05). Interestingly, women showed a significantly higher detection rate of mCRP than men (21.0 vs 3.8%, p=0.045). No association of mCRP levels with any of the remaining studied parameters was observed.

### 3.2 Low levels of systemic FH are associated with intermediate AMD

**Figure 1:**
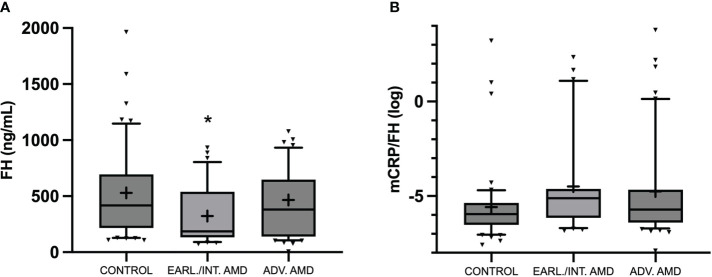
Box and whisker plot representing serum circulating FH levels (ng/mL) **(A)**, and mCRP/FH (log) **(B)** in control, early/intermediate AMD, and advanced AMD. Median values are highlighted by solid lines and mean values by “+”. Statistical analysis was conducted using Kruskal-Wallis (*P<0.05 vs. control).

Systemic FH levels were significantly lower in the early/intermediate AMD group (186.47 (72.11-931.78) ng/mL) compared to the advanced AMD (380.27 (5.39–2680) ng/mL) and the healthy control (415.23 (106.13-1962.21) ng/mL) groups (p=0.039) (**Figure 1A**). In addition, the mCRP/FH ratio (log) was higher in the early/intermediate group (-5.15 (-6.84–2.35)) than in the advanced AMD (-5.72 (-7.89–3.79)) and control groups (-5.95 (-7.58–3.22) (p=0.031) (**Figure 1B**).

Low FH levels (<200 ng/mL) were significantly associated with the presence of drusen and pigmentary changes (62.8% vs. 37.2%, p=0.002). No association was found between FH and sex (p=0.235), age (p=0.403), visual acuity (p=0.873), foveal thickness (p=0.653), and macular volume (p=0.990). Instead, reduced FH levels were associated with increased choroidal thickness (228.90 ± 62.69 µm vs. 183.21 ± 62.86 µm, p=0.003) (**Table 2**).

**Table 2:** Bivariate analysis of FH according to >200 ng/mL level category cut.

	FH > 200				
	No		Yes		p-value
	Total	%	Total	%	
*Age, years (mean ± SD)*	77.05 ± 8.44		78.45 ± 7.15		0.403
*Women (N)*	30	48.4	32	51.6	0.235
*Men (N)*	9	34.6	17	65.4	
*BCVA, logMAR* *Median (min.- max.)*	0.30 (0-3)		0.30 (0-3)		0.957
*IOP, mmHg (mean ± SD)*	16.87 ± 2.49		15.53 ± 3.16		0.036*
*Drusen, pigmentary changes (%)*	62.8		37.2		0.002*
*Atrophy, active NVM, fibrosis, PED (%)*	27.3		72.7		
*Foveal thickness*, µm *Median ± IQR (min.- max.)*	245 (132 - 478)		251 (93 - 479)		0.653
*Macular volume*, mm^3^ (*Mean ± SD*)	9.81 ± 0.88		9.81 ± 0.88		0.990
*Choroidal thickness*, µm (*Mean ± SD*)	228.90 ± 62.69		183.21 ± 62.86		0.003*

SD, Standard deviation; BCVA, best corrected visual acuity; IQR, Interquartile Q1-Q3 range; Max., maximum; Min., minimum; IOP, intraocular pressure; NVM, neovascular membrane; PED, pigment epithelium detachment. Bold values are statistically significant values.

### 3.3 Impact of Y402H SNP on systemic levels of CRP conformations and FH

The distribution of the CRP-FH axis was analyzed according to the rs1061170 SNP in CFH gene. As seen in **Table 3**, no differences were observed on mCRP, pCRP, and FH systemic levels. Interestingly, significant differences were found in the mCRP/FH ratio, being higher in individuals with the risk “CC” variant (p=0.048).

**Table 3:** Distribution of mCRP, pCRP, FH, mCRP/pCRP and mCRP/FH across the rs1061170 genotype.

		SNP Y402H		
Biomarkers median (min-max)	TT	CT	CC	p-value
**mCRP** (ng/mL)	1 (1-645.5)	1 (1-5825)	1 (1-3010)	0.234
**pCRP** (µg/mL)	1.68 (0.055-18.17)	1.42 (0.06-15.64)	1.63 (0.30-9.78)	0.700
**FH** (ng/mL)	561.16 (85.63-1326)	354.25 (72.24-1959)	162.39 (89.93-1075)	0.153
**Ratio mCRP/pCRP (log)**	-0.28 (-2.75-6.28)	0.15 (-2.75-8.83)	0.82 (-2.28-7.12)	0.150
**Ratio mCRP/FH (log)**	-6.27 (-7.19-1.83)	-5.24 (-7.58-3.79)	-4.91 (-6.91-3.22)	0.048*

mCRP, monomeric C-reactive Protein; pCRP, pentameric C-reactive Protein; FH, complement Factor H; SNP, single nucleotide polymorphism. Bold values are statistically significant values.

## 4 Discussion

The contribution of local complement activation and immune mediated processes to the pathophysiology of AMD has gained considerable interest in recent years. Nevertheless, there is an unmet need to identify systemic biomarkers associated with the local status of the disease, which could potentially help us to stratify and select patients for suitable tailored therapies. We evaluated the potential of systemic mCRP, the proinflammatory conformation of CRP, and its regulator FH as potential biomarkers of severity in AMD patients.

CRP is a dynamic protein that undergoes conformational changes upon activation in inflammatory microenvironments between pentameric (pCRP) and monomeric (mCRP) forms. Although pCRP is the circulating form routinely tested for clinical purposes, mCRP is the proinflammatory conformation of CRP. Of note, mCRP induces inflammation and barrier disruption in RPE (14) and choroidal endothelial cells (19) and has been localized in drusen (20). Thus, it could therefore represent a more accurate marker of disease. However, given its insoluble nature, mCRP is not easily detected in serum and there are no commercially available assays.

In our work we adapted the ELISA protocol described by Zhang et al. to determine the circulating levels of mCRP as this protocol avoids cross-reactivity with pCRP by using a capture antibody that specifically recognizes mCRP (clone CRP-8) (17). Using this technique circulating mCRP was detected in less than 15% of our cohort. Notably, mCRP was only detected in 9.8% of control subjects, 15.1% of patients with intermediate AMD, and 18.2% of patients with advanced AMD. Yet, AMD patients tended to present higher levels of mCRP than healthy subjects, especially in the advanced AMD group. These observations are conceivable with a low-grade chronic inflammation that is not translated into high levels of circulating mCRP. Indeed, because mCRP dissociates from pCRP, higher levels of pCRP may be needed to result in detectable levels of circulating mCRP. In fact, we found increased mCRP levels to be significantly associated with increased pCRP levels. In this line, systemic mCRP has been found significantly increased in acute inflammatory diseases with higher pCRP levels such as acute myocardial infarction (17) or COVID-19 (21). Most AMD patients in our cohort, as in general population, were female. Studies on gender predilection have reported a female preponderance for AMD, probably related to a longer life expectancy (22). Interestingly, women showed a higher detection rate of mCRP than men, which may reflect an enhanced proinflammatory microenvironment. These observations should be validated in a larger cohort but may be relevant as it has been shown that women with neovascular AMD present a significant increase in pCRP levels compared with control women and with men with neovascular AMD, which was linked to the A69S genotype of *ARMS2* gene (23).

Age and disease progression time was higher in the advanced AMD group, which is consistent with AMD chronic and progressive course. As expected, BCVA was preserved in the control group, mildly affected in the early/intermediate AMD group and severely affected in the advanced AMD group, as deterioration of retinal layers occurs. As predictable, macular atrophy, fibrosis, NVM and pigment epithelium detachment were found in advanced AMD, whereas drusen and pigmentary changes were found in both AMD groups (1). Macular volume was significantly lower in the advanced AMD group where macular atrophy was present in the fundoscopy exam in 12 patients. Likewise, foveal thickness showed a trend to be slightly lower in both AMD groups. Choroidal thickness values were also lower in the advanced AMD group, where both presence of atrophy and treatment with intravitreal anti-vascular endothelial growth factor, which is indicated at this stage, are known to be associated with thinner choroid (24).

While choroidal thickness was found here to be increased in early/intermediate patients with higher CRP levels, previous research studies have related increased CRP levels with thinner choroid (25), thus supporting a role for CRP as an inflammation biomarker of AMD progression. However, such studies did not differentiate among AMD stages and could be explained by the presence of atrophy or previous treatment in advanced AMD. Research in this field goes further in OCT analysis and includes parameters as drusen characteristics, hyperreflective foci, presence of pigmentary hyperreflective material in the retina, RPE and outer retina atrophic lesions or/and subsidence of the outer plexiform layer and inner nuclear layer as AMD progression biomarkers (26, 27).

In our study, patients with AMD presented complement FH levels significantly lower than controls, and particularly patients with early/intermediate AMD presented the lowest FH levels. There are conflicting results regarding FH levels in AMD as some studies have reported FH levels to be decreased in AMD patients (4, 5, 28), whereas other studies with larger AMD cohorts have found similar levels to controls (29–31). These studies included AMD patients with advanced AMD which could explain the differences as we observed FH levels to be particularly decreased in patients with early/intermediate AMD. Certainly, one study that evaluated complement activation status in intermediate AMD also observed reduced FH levels (32). In their study, Lynch et al. also observed increased complement activation. Indeed, we also observed that low FH levels were associated with the presence of drusen and pigmentary changes, which were mostly present in patients with early/intermediate AMD. Whether the inflammatory status of early/intermediate AMD then switches into a more atrophic phenotype requires further research. In our cohort, reduced FH levels were associated with increased choroidal thickness. This is not fully consistent with FH levels being lower in the AMD groups, which tend to have thinner choroid. This could be justified by a natural variability of this parameter among subjects (33).

On the other hand, FH levels may also be related to variants in the *CFH* locus (29, 30), as shown in several studies. Indeed, a recent study reported that AMD patients carrying rare or low-frequency variants in the *CFH* gene presented lower FH levels in conjunction with increased complement activation (34). Indeed, we did not test low frequency variants of the CFH locus. We only tested the Y402H *CFH* polymorphism, and no associations with FH levels were observed. Interestingly, the Y402H polymorphism in CFH gene significantly affected mCRP/FH ratio, being higher in patients carrying the risk variant. Whether and how the impaired binding of FH to mCRP in patients carrying the risk variant may lead to increased mCRP/FH ratio requires further research.

The relative contribution of local and systemic inflammation and complement activation to the pathophysiology of AMD is yet to be fully elucidated. It is likely that both local and systemic complement contribute and compound one another in AMD. Specifically, FH is produced by the liver but also in the RPE, and the fact that the “risk” Y402H polymorphism does not affect circulating FH levels could imply a stronger contribution of local FH. In this regard, Khandhadia et al. showed that AMD was not associated with modified hepatic FH production, further supporting the importance of locally produced FH in AMD (35). Our results suggest that in the early stages of the disease, systemic FH could play a relevant role. Furthermore, the follow-up of our cohort of intermediate AMD patients could also shed light not only on the role of FH as a player but also as a prognostic biomarker of disease progression. Moreover, the increased mCRP/FH ratio, especially in the intermediate AMD group, also supports the contributing role of a systemic proinflammatory environment in early stages of the disease.

Despite the limitations of our study mainly due to the limited sample size, our results support the role of systemic inflammation in the early stages of AMD. In particular, low levels of circulating FH and increased mCRP/FH ratios in early/intermediate AMD patients. Such findings should be validated in larger cohorts since, if confirmed, they could provide useful biomarkers to stratify patients, produce a risk score and enhance individualized therapies.

## Data availability statement

The original contributions presented in the study are publicly available. This data can be found here: https://github.com/jvannesa/Factor-H-Age-Related-Macular-Degeneration.

## Ethics statement

The studies involving humans were approved by Hospital Clínic of Barcelona Institutional Review Board (IRB) (HCB/2017/0558). The studies were conducted in accordance with the local legislation and institutional requirements. The participants provided their written informed consent to participate in this study.

## Author contributions

LG: Data curation, Investigation, Writing – original draft. MF: Formal analysis, Investigation, Supervision, Writing – review & editing. BE: Data curation, Investigation, Writing – review & editing. BR: Writing – review & editing. JZ: Investigation, Writing – review & editing, Data curation. SA: Investigation, Methodology, Writing – review & editing. FS: Investigation, Writing – review & editing, Methodology. JB: Writing – review & editing. FL: Writing – review & editing, Investigation, Resources, Supervision, Validation. MD: Investigation, Writing – review & editing. AS: Data curation, Formal analysis, Writing – review & editing, Methodology. PD: Writing – review & editing. JI: Writing – review & editing, Investigation. OV: Formal analysis, Writing – review & editing, Data curation. AA: Funding acquisition, Supervision, Writing – review & editing. AF: Conceptualization, Supervision, Writing – review & editing. BM: Conceptualization, Formal analysis, Funding acquisition, Investigation, Methodology, Supervision, Writing – original draft, Writing – review & editing.
